# Computer-assisted rehabilitation system in the use of motor function recovery: A protocol for scoping review

**DOI:** 10.1371/journal.pone.0326865

**Published:** 2025-07-01

**Authors:** Jun Fan, Xuqiang Wei, Yue Yao, Yibang Jiang, Cankun Xin, Ling Feng

**Affiliations:** 1 Department of Rehabilitation Medicine, Yueyang Hospital of Integrated Traditional Chinese and Western Medicine, Shanghai University of Traditional Chinese Medicine, Shanghai, People’s Republic of China; 2 Acupuncture Anesthesia Clinical Research Institute, Yueyang Hospital of Integrated Traditional Chinese and Western Medicine, Shanghai University of Traditional Chinese Medicine, Shanghai, People’s Republic of China; Huashan Hospital Fudan University, CHINA

## Abstract

**Backgrounds:**

Motor dysfunction, a prevalent sequela of neurological disorders such as stroke, Parkinson’s disease, and cerebral palsy, profoundly compromises patients’ capacity to perform daily activities and participate in social interactions. To address this challenge, computer-assisted rehabilitation systems (CARS) have emerged as innovative tools for facilitating motor function recovery. However, the rapid proliferation of diverse CARS modalities—encompassing novel technical approaches, interdisciplinary integrations, and heterogeneous clinical applications—has resulted in a fragmented and poorly delineated research landscape. This scoping review protocol aims to systematically map the current evidence, clarify conceptual boundaries, identify key research themes, and highlight critical knowledge gaps within the CARS field.

**Methods:**

Following PRISMA-ScR guidelines, a comprehensive search across English (PubMed, Embase, Web of Science) and Chinese (CNKI, Wanfang, VIP, SinoMed) databases (inception to May 2025) will retrieve peer-reviewed studies using controlled vocabulary (MeSH: Rehabilitation/methods) and keywords (“computer-assisted rehabilitation system*”). After deduplication (EndNote X9) and manual verification, two reviewers will independently screen titles/abstracts and full texts, resolving discrepancies via third-party arbitration. Data extraction will categorize studies into study characteristics (design, population), technical specifications (sensors, AI), and clinical contexts (outcome measures, motor domains). Quantitative synthesis will map publication trends, geographic distributions, and methodological profiles using PRISMA diagrams and heatmaps. Thematic analysis will identify dominant research clusters (e.g., robotics, VR) and interdisciplinary linkages. Results will be disseminated via interactive evidence maps and narrative summaries emphasizing clinical translation. Any protocol deviations will be explicitly documented to ensure methodological transparency.

**Discussion:**

This review will synthesize the heterogeneous CARS field into a structured framework, guiding future research prioritization and clinical implementation. By delineating technical innovations, clinical efficacy, and knowledge gaps, findings aim to optimize rehabilitation strategies for neurological populations.

**Detail of this review project can be found in Open Science Framework:** https://doi.org/10.17605/OSF.IO/HXDT8.

## 1. Introduction

Motor dysfunction, a prevalent consequence of neurological disorders, such as stroke [1], Parkinson’s disease [2], and cerebral palsy [3], significantly impairs patients’ ability to perform in daily activities and social interactions. The prognosis of these disorders is influenced by various factors, including age and genetics [4]. A recent study [5] has pointed out that with the high incidence of such diseases, an increasing number of patients are expected to develop motor impairments. Effective rehabilitation interventions are crucial for slowing disease progression, improving functional outcomes, and reducing the incidence of complications [2,6].

Emerging technologies show significant potential to enhance the recovery of physical functions. Recent advancements have introduced CARS as innovative tools for motor training and posture/behavior analysis, offering improved therapeutic prospects [7]. Substantial evidence indicates that CARS can modulate neurobiological mechanisms underlying motor recovery. For instance, motion-capture systems facilitate repetitive task-specific training to enhance cortical reorganization, while motor platforms generate controlled perturbations that may modulate neuronal excitability via mechanosensitive ion channels or acoustic pathways [8]. Virtual reality (VR) has been shown to potentially increase brain-derived neurotrophic factor (BDNF) expression by promoting multisensory integration and cognitive engagement [9]. CARS enables highly repetitive, task-oriented training with multimodal feedback (e.g., visual and auditory), supporting functional recovery through neuroplasticity mechanisms. Emerging studies indicate that computer-assisted techniques are relevant for precision medicine and high-throughput simulation, though they also highlight the need for further exploration of how biochemical, mechanical, and structural signals can be integrated to achieve precise modulation [10,11]. VR systems offer innovative opportunities for motion training and the study of human posture and behavior. Some systems integrate sophisticated motion capture technology with hydraulic and mechanical actuation. This integration enables dynamic adjustments to walking patterns and real-time motion tracking. The result of this integration is the capability for detailed kinematic and biomechanical analysis. The integration of fUS-BCI into CARS may mechanistically link technological interventions to synaptic remodeling [12]. For example, haptic feedback integration enhances proprioceptive input and may facilitate spinal cord-level synaptic remodeling through long-term potentiation mechanisms [13]. While BDNF upregulation enhances synaptic plasticity, PRMT1-dependent mitochondrial integrity may represent a parallel pathway for CARS-mediated mitigation of motor neuron degeneration [14].

A prominent example is the Computer Assisted Rehabilitation Environment (CAREN) developed by MOTEK Medical (Amsterdam, Netherlands). This integrated system combines motion capture technology with a six-degree-of-freedom platform, force plates and treadmill components [15,16]. The system allows clinicians to administer controlled visual and physical perturbations during rehabilitation, requiring patients to make dynamic gait adjustments. CAREN’s modular design supports customizable virtual reality immersion levels, while its real-time motion tracking capability enables detailed frame-by-frame kinematic and biomechanical analysis [17]. Clinical studies have validated CAREN’s efficacy as an adjunct to conventional rehabilitation, particularly for improving postural instability, muscle rigidity, and balance deficits [18–21]. Nevertheless, the neurophysiological mechanisms underlying these technology-mediated functional improvements remain incompletely characterized. Although computer-assisted systems show promise for big data analytics in precision medicine, their clinical implementation faces challenges in data standardization and requires interdisciplinary collaboration to maximize benefits for both clinicians and patients.

Although there are a few reviews on the use of CARS, the topics are mainly focused on the restoration of cognitive function or the use of evaluation of treatment [7,22,23]. The existing literature also focuses mainly on clinical results rather than on how the technological components (e.g., VR immersion, feedback delay) are related to molecular pathways of the living cell (support for axonal regeneration or regulation of ROS). However, the expansion of CARS modalities—driven by technological innovation, interdisciplinary convergence, and diverse clinical use cases—has paradoxically fragmented the field, obscuring its conceptual boundaries. To address this gap, scoping review systematically maps and evaluates existing evidence on CARS for motor rehabilitation, analyzing their efficacy in functional recovery and broader impacts on patients’ daily living and societal engagement is urgently needed. Through rigorous analysis of technical-clinical interdependencies, this work seeks to establish an evidence-based framework to optimize rehabilitation protocols and guide targeted innovation in neurorehabilitation technologies.

## 2. Methods

### 2.1. Study registration

This scoping review protocol follows the PRISMA-ScR guidelines [24] and has been prospectively registered on the Open Science Framework on December 6, 2024 (OSF: https://osf.io/hxdt8), with iterative updates archived for transparency. Adopting the Arksey and O’Malley framework [25], we will implement five methodological phases: (a) defining research questions, (b) identifying evidence sources, (c) study selection, (d) data extraction and charting, and (e) evidence synthesis. The workflow will commence on 1 May 2024, with phased completion over 6 months. Key methodological components—including rationale, search strategy, inclusion criteria, data synthesis, limitations, and protocol transparence—are systematically detailed in the main text. All protocol versions, including open peer review feedback, are permanently accessible via OSF to ensure reproducibility. We updated the registration on May 20, 2025. The study involves secondary data analysis and does not require ethical approval.

### 2.2. Review questions

Primary questions: a) What methodological approaches characterize CARS research in motor rehabilitation? b) What are the scope and defining features of published CARS literature in this domain?
Secondary questions: a) What are the main gaps found in the existing literature and propose issues that future researches may need to address. b) What are the mainstream intervention durations and details and how to evaluate longitudinal treatment effects.

### 2.3. Searching strategy

An exploratory Medline search informed the development of a structured search syntax, which combines two conceptual domains using Boolean operators: 1) terms related to the study population and rehabilitation outcomes, including “neurorehabilitation,” “gait rehabilitation,” “motor cortex activation,” “gait kinematics,” “lower limb rehabilitation,” “upper limb rehabilitation,” and “motor recovery”; 2) terms describing intervention technologies, including “computer-assisted” and “virtual reality.” Seven multidisciplinary databases (Medline, Embase, WOS, CNKI, VIP, Wanfang, SinoMed) will be searched from inception through May 2025 by a trained reviewer. The strategy employs controlled vocabulary (MeSH terms), free-text keywords, truncation, and proximity operators (full syntax in S1 Appendix). No language restrictions apply.

### 2.4. Data selection

**Fig 1:**
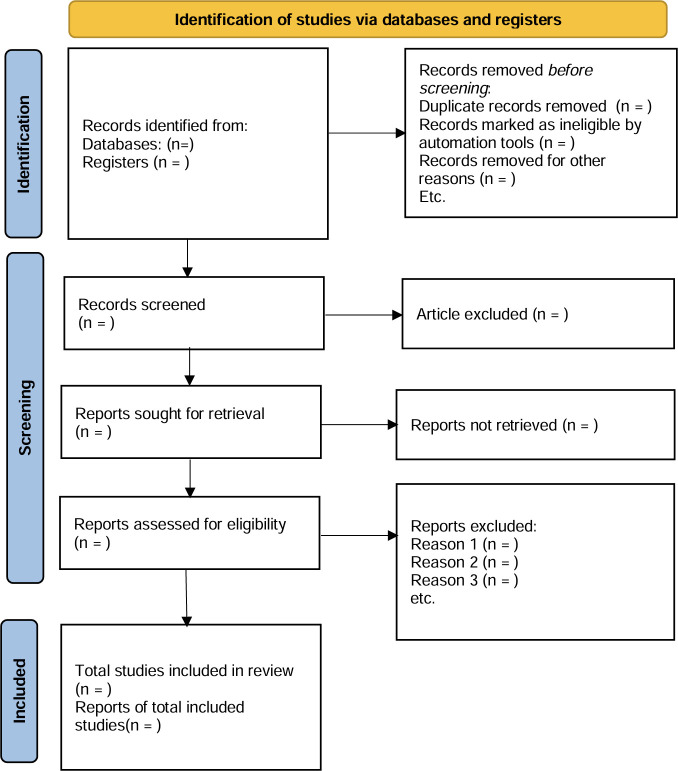
The PRISMA 2020 flow diagram^*^ for reporting screening results [27]. *: Adapted from Page MJ, et al. PRISMA 2020 flow diagram (https://doi.org/10.1136/bmj.n71), distributed under the terms of the Creative Commons Attribution (CC BY 4.0) license (https://creativecommons.org/licenses/by/4.0).

Two independent reviewers (J Fan and YB Jiang) will conduct blinded screening in EndNote X9. Reviewers will independently screen titles/abstracts and full texts while blinded to author names, institutions, and publication journals using predetermined inclusion/exclusion criteria. After the preliminary assessment, the full text of the selected literature will be evaluated. Disputes will be decided by a third reviewer (XQ Wei). The process of the review will be reported in the PRISMA flow diagram (Fig 1). The pilot calibration with 50 randomly selected records and the inter-rater reliability monitoring will be evaluated via Cohen’s κ (target κ > 0.80) [26]. In order to further validate the reproducibility of the screening results, the third researcher will reassess 20% of the screening records. This quality control measure will be applied at both title/abstract and full-text screening stages to minimize selection bias.

### 2.5. Inclusion and exclusion criteria

#### 2.5.1. Eligibility criteria.

Studies meeting all following conditions will be included:

a) Study design: Original research articles (RCTs, cohort studies, case reports) or protocol papers;
b) Population: Adults (≥18 years) with neurologically confirmed motor dysfunction (stroke, Parkinson’s disease, multiple sclerosis);
c) Intervention: Computer-assisted rehabilitation systems (CARS) as primary therapeutic modality;
d) Accessibility: Full-text availability through institutional subscriptions or open access.

#### 2.5.2. Exclusion criteria.

a) Content type: Animal studies, computational models, algorithm development, or technical specifications;
b) Population focus: Pediatric cases, healthy subjects, military personnel, or non-motor outcomes;
c) Publication format: Conference abstracts, opinion pieces, or non-systematic reviews;

Data limitations: Unclear diagnostic criteria or duplicate datasets.

### 2.6. Data extraction

Two independent reviewers will conduct standardized data extraction using a pre-piloted template in Microsoft Excel (v2021), with discrepancies resolved through consensus or third-party adjudication. Extracted variables are organized into four domains (Table 1). Following quality verification, all data will be synthesized into an interactive evidence matrix (S1 Table). Quantitative variables will be visualized through heatmaps to demonstrate intervention-outcome associations, while qualitative findings will be thematically mapped using NVivo 14.

**Table 1:** Draft data extraction table.

Domain	Data Elements
**Study Characteristics**	Authors, publication year, country, design (RCT/cohort/case), sample size
Patient Profile	Age, diagnosis, disease duration, baseline functional status
**Intervention Parameters**	CARS technology type (e.g., VR/robotics), session duration, treatment frequency
**Outcome Metrics**	Clinical scales (e.g., Fugl-Meyer), biomechanical parameters, biomarkers, longitudinal outcomes (≥6 months)

### 2.7. Data analysis and Evidence Synthesis

This scoping review will employ a PRISMA-ScR-compliant framework incorporating three analytical dimensions:

I) **Multimodal Data Integration**
  - **Study profiles**: Tabulated summaries of design characteristics (RCT/cohort/case), intervention parameters (technology type, session duration), and outcome metrics (clinical/biomechanical/biological) (S1 Table)
  - **Disease mapping**: Anatomical distribution of motor impairments visualized via schematic body diagrams with layered annotations (acute/chronic phase, upper/lower limb involvement)
  - **Mechanistic insights**: Critical biological metrics (serum biomarkers, fMRI connectivity, EMG patterns) analyzed through interactive heatmaps displaying biomarker-outcome correlations
II) **Conflict Resolution Protocol**
  a) **Stratification strategies**:
    - *Thematic*: Group studies by rehabilitation paradigm (e.g., task-specific vs. compensatory training)
    - *Subgroup*: Stratification by disease chronicity (<6 months vs. > 6 months post-onset) and treatment intensity (≥3 sessions/week)
  **b) Evidence weighting**: GRADE-adapted certainty assessment for conflicting outcomes (e.g., balance vs. spasticity measures)
III) **Translational Framework Development**
  a) **Clinical decision matrix**: Evidence-to-practice tool aligning CARS technologies with patient characteristics (age, impairment severity, comorbidities)
  b) **Research prioritization**: Gap analysis visualized through radar plots comparing evidence density across PICO elements

All synthesized data will be disseminated via an open-access interactive evidence map (https://osf.io/hxdt8) allowing real-time filtering by:

- Technological maturity level (prototype/validated/commercialized)
- Outcome significance (clinical vs. statistical improvement)
- Biomarker mechanistic relevance (ROS regulation, neuroplasticity indices)

### 2.8. Study status and timeline

This protocol outlines a planned scoping review with the following proposed timeline:

1) **Literature search completion**: May 31, 2025.
2) **Literature screening completion:** June 30, 2025.
3) **Data extraction**: To commence following screening, with anticipated completion by August 31, 2025.
4) **Data synthesis and manuscript preparation**: October 31, 2025.

**Fig 2:**
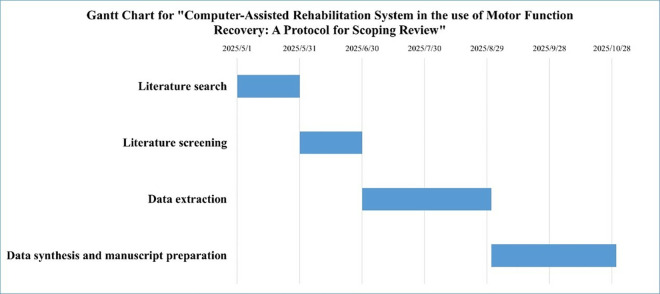
Timeline of the scoping review.

No results or preliminary findings are available at this stage. The project timeline is presented in a Gantt chart (Fig 2). To mitigate potential delays, we implemented the following contingency protocols:

1) **Phased retrieval**: Core databases (MEDLINE/CNKI) will be prioritized, with progress monitored daily.
2) **AI-assisted translation**: Translations will be performed using DeepL (www.deepl.com), followed by verification from bilingual researchers.
3) **Resource allocation**: Additional reviewers will be assigned if translation delays occur.

## 3. Discussion

This scoping review systematically addresses the critical need to consolidate evidence on CARS for motor recovery, particularly given the current heterogeneity in technological implementations and fragmented understanding of underlying mechanisms. This review will synthesize multi-dimensional diagnostic and therapeutic data to develop a comprehensive, evidence-based framework. By mapping anticipated studies across seven databases, we will establish a foundational taxonomy linking technical specifications (e.g., VR latency thresholds <100ms) to clinical outcomes (e.g., Fugl-Meyer improvements ≥5 points), while identifying understudied areas such as cerebellar ataxia rehabilitation.

Although quality assessment is not standard in a scoping review [28], we will discuss its implementation to strengthen the credibility of our findings, particularly for audiences requiring robust evidence. Scoping reviews typically identify, present and describe relevant characteristics of the included sources of evidence. Our review will consider the study designs included and select appropriate tools (e.g., ROB2.0 for RCTs, Newcastle-Ottawa Scale for non-randomized studies, etc.) while adhering to PRISMA-ScR reporting standards to ensure transparency. We will also assess the impact of low-quality evidence on conclusions and identify key areas requiring validation through high-quality RCTs,

This scoping review aims to advance CARS by finding several key pathways. First, technological innovations will focus on enhancing existing devices through the development of wearable and miniaturized portable platforms to better support tele-rehabilitation and home-based therapeutic interventions. Second, biomarker-driven personalization will identify pathognomonic biomarkers across neurological pathologies, enabling tailored therapeutic regimens aligned with individual neuroplasticity profiles. Finally, interoperable CARS architectures will integrate multidimensional data to establish transdiagnostic rehabilitation databases. Such infrastructure will enable mechanistic analysis of treatment responses through computational modeling of cross-pathology motor recovery patterns. Collectively, these pathways will establish a translational framework for precision rehabilitation ecosystems, integrating engineering innovations, clinical neuroscience, and health informatics.

We propose the following testable hypotheses and corresponding experimental approaches for future mechanistic studies. First, integration of biomechanics with neural control could elucidate how CARS integrates motor control. This could be investigated through: (1) combined motion capture and electromyography in patients, (2) high-density surface EMG to quantify neuromuscular coordination patterns during CARS interventions, and (3) virtual environment simulations in animal models to monitor cerebellar and basal ganglia activity. For molecular mechanism, research can be focused on identifying specific pathways involved. Potential mediators include neurotrophic factors (e.g., BDNF) and inflammatory markers. Specifically, CARS-mediated motor recovery may involve BDNF upregulation, which could be examined through: (1) longitudinal analysis of serum and cerebrospinal fluid samples in animal models to correlate BDNF levels with functional recovery, (2) conditional BDNF knockout mice subjected to CARS-like training protocols to establish causality, and (3) in vitro neuronal cultures with controlled mechanical/electrical stimulation to monitor gene expression changes. Future directions should also explore whether CARS-induced long-term effects involve epigenetic modifications and identify genetic subpopulations with preferential treatment responses.

The study aims to systematically evaluate existing clinical evidence in computer-assisted rehabilitation for motor dysfunction. The research has two primary objectives: (1) to offer evidence-based decision support for clinical practitioners, and (2) to identify current theoretical gaps and technological limitations in the field. By establishing an interdisciplinary knowledge framework, the study seeks to facilitate the translation of computer-assisted rehabilitation technologies from experimental research to standardized clinical implementation.

This scoping review will synthesize evidence exclusively from publicly accessible scientific repositories, thereby precluding the need for institutional review board (IRB) approval under current ethical guidelines for secondary data analyses [26]. The findings will be disseminated through a peer-reviewed journal publication, providing a comprehensive literature synthesis. By employing structured knowledge translation frameworks, this work aims to bridge the gap between innovation and clinical practice in rehabilitation engineering. The findings may inform value-based rehabilitation protocols across diverse care settings by establishing clinical meaningful linkages between technological capabilities and patient functional outcomes.

## 4. Strengths and limitations

The proposed review will be the first scoping review of CARS for motor function recovery. Multi-database research which ensures comprehensiveness of the research will be conducted to explore in detail the current status and evidence of this rehabilitation technique in clinical trials. This protocol includes a systematic search and adheres to a rigorous, established research framework for the selection and reporting of studies. This review will not include a meta-analysis of prediction performance. There is no evaluation of research quality in this article and its purpose is to identify a wide range of literature. We will not thoroughly evaluate the potential for bias and the quality of evidence incorporated in the study, as the objective is to identify and summarize areas of research that are lacking and potential avenues for further investigation.

Through a comprehensive analysis of existing literature and application of PRISMA-ScR framework, the study will provide a comprehensive overview of the application of CARS in motor function recovery, and point out the direction of future research. This will not only help advance science in the field, but also provide guidance for clinical practice.

## Supporting information

S1 Appendix The search strategy for both English and Chinese databases. (DOCX)

S1 Table Interactive evidence matrix for data extraction. (XLSX)

S1 Checklist PRISMA-P 2015 Checklist. (DOCX)
