# Supplementary material for: Computer-assisted rehabilitation system in the use of motor function recovery: A protocol for scoping review
Source: PLoS One. 2025 Jul 1;20(7):e0326865. doi: 10.1371/journal.pone.0326865 (PMC12212561; doi:10.1371/journal.pone.0326865)
Supplement: S1 Appendix — (DOCX) [file pone.0326865.s001.docx]

| **Supplementary Table 1** **\|** The search strategy for English databases. | | |
| --- | --- | --- |
| **Database** |  | **Strategy** |
| **EMBASE** |  | ('neurorehabilitation':ti,ab,kw OR 'gait rehabilitation':ti,ab,kw OR 'motor cortex activation':ti,ab,kw OR 'gait kinematics':ti,ab,kw OR 'lower limb rehabilitation':ti,ab,kw OR 'upper limb rehabilitation':ti,ab,kw OR 'motor recovery':ti,ab,kw) AND ('computer assisted':ti,ab,kw OR 'virtual reality':ti,ab,kw) |
| **Web of science** |  | 1: ALL=("computer assisted")  2: ALL=("virtual reality")  3: #1 OR #2  4: ALL=("neurorehabilitation")  5: ALL=("gait rehabilitation")  6: ALL=("motor cortex activation")  7: ALL=("gait kinematics")  8: ALL=("Lower Limb Rehabilitation")  9: ALL=("Upper Limb Rehabilitation")  10: ALL=("Motor Recovery")  11: #4 OR #5 OR #6 OR #7 OR #8 OR #9 OR #10  12: #11 AND #3 |
| **Medline** |  | ("computer assisted"[All Fields] OR "virtual reality"[All Fields]) AND ("neurorehabilitation"[All Fields] OR "gait rehabilitation"[All Fields] OR "motor cortex activation"[All Fields] OR "gait kinematics"[All Fields] OR "Lower Limb Rehabilitation"[All Fields] OR "Upper Limb Rehabilitation"[All Fields] OR "Motor Recovery"[All Fields]) |

| **Supplementary Table 2 \| The search strategy for Chinese databases.** | |
| --- | --- |
| **Database** | **Strategy** |
| **CNKI** | **SU %= （“计算机辅助” + “虚拟” + “电脑辅助”） AND SU %= (“上肢训练” + “下肢治疗” + “运动康复” + “神经康复” + “步态锻炼” + “运动皮质激活”)** |
| **Wangfang Data** | **题名或关键词:(计算机辅助 OR 虚拟 OR 电脑辅助) and 题名或关键词:(运动康复 OR上肢康复 OR 下肢康复 OR 平衡功能 OR 步态康复 OR 神经康复 OR 运动皮质激活)** |
| **VIP** | [**(((题名或关键词=计算机辅助 OR 题名或关键词=虚拟) OR 题名或关键词=电脑辅助) AND (((((题名或关键词=运动康复 OR上肢康复 OR 题名或关键词=下肢康复) OR 题名或关键词=平衡功能) OR 题名或关键词=步态康复) OR 题名或关键词=神经康复) OR 题名或关键词=运动皮质激活))**](http://qikan.cqvip.com/Qikan/search/index?LngMySearHistoryIdGuid=2aa639fa-217b-4334-a09f-2e8ce41dd182&from=Qikan_Article_History) |
| **Sinomed** | [**( "计算机辅助"[常用字段:智能] OR "虚拟"[常用字段:智能] OR "电脑辅助"[常用字段:智能]) AND( "运动康复"[常用字段:智能] OR "上肢康复"[常用字段:智能] OR "下肢康复"[常用字段:智能] OR "平衡功能"[常用字段:智能] OR "步态康复"[常用字段:智能] OR "神经康复"[常用字段:智能] OR "运动皮质激活"[常用字段:智能])**](javascript:toDoRelimitSearch();) |
